# Salvage of Failed Local and Regional Flaps with Porcine Urinary Bladder Extracellular Matrix Aided Tissue Regeneration

**DOI:** 10.1155/2013/917183

**Published:** 2013-09-26

**Authors:** Gregory J. Kruper, Zachary P. VandeGriend, Ho-Sheng Lin, Giancarlo F. Zuliani

**Affiliations:** ^1^Department of Otolaryngology, Head and Neck Surgery, Wayne State University School of Medicine, 4201 St. Antoine, 5E-UHC, Detroit, MI 48201, USA; ^2^Department of Otolaryngology, Head and Neck Surgery, Wayne State University School of Medicine and Karmanos Cancer Institute, 4201 St. Antoine, 5E-UHC, Detroit, MI 48201, USA; ^3^Division of Facial Plastic and Reconstructive Surgery, Department of Otolaryngology, Head and Neck Surgery, Wayne State University School of Medicine and Karmanos Cancer Institute, 4201 St. Antoine, 5E-UHC, Detroit, MI 48201, USA

## Abstract

Local and regional flap failure can be a major complication in head and neck surgery, which continue to be prevalent for a number of reasons including poor flap design, improper surgical technique, and poor tissue vascularity. Dealing with these failures can be quite difficult. Surgical debridement, flap revisions, and complex wound regimens are necessitated to reestablish appropriate tissue coverage. Traditional use of wet to dry dressing to enable proper wound granulation and possible closure with additional flaps or skin grafts is a laborious process. Such treatments place great time burdens on the patient, physicians, and nurses. Because the face and neck possess a complex three-dimensional topography, wound dressings are inherently complex to design and change. Many patients also require postoperative treatments such as radiation and chemotherapy to treat aggressive malignancies, and delay in wound healing leads to a delay in adjuvant treatment. Recently, advances in regenerative medicine, specifically xenogeneic extracellular matrix compounds, have been shown to promote tissue growth while limiting scar tissue formation (Badylak 2004). To our knowledge, this paper is the first case series using the porcine extracellular matrix bioscaffold (MatriStem ACell, Columbia, MD, USA) to salvage flaps with extensive wound breakdown on the face and neck.

## 1. Introduction

 Local and regional flap survival depends on proper design, meticulous surgical technique, and factors intrinsic to the patient. Modern head and neck surgeons see many patients who have extensive medical co-morbidities such as diabetes, collagen vascular disorders, and peripheral vascular disease that make the survival of local random flaps and even pedicled flaps more tenuous [1]. In addition, smoking, malnutrition, and fluid overload during surgery may increase the likelihood of flap failure [2]. Flap failure increases the length of surgical stay, places additional economic burden on the health care system, and can delay adjuvant therapy in cases of malignancy. Additional medical costs related to flap salvage are mostly found in the vascular surgery and chronic wound literature and estimated conservatively at $30,000 per case [3]. 

 To understand flap failures, one must understand wound healing. Initial injury to skin results in disruption of blood vessels and extravasation of blood cell contents. Aggregation of platelets, fibrinogen, fibrin, and fibronectin forms a blood clot which reestablishes homeostasis. This clot provides the initial matrix for cell attachment and migration. Platelets release platelet derived growth factor, transforming growth factor beta, and epidermal growth factor. These cytokines provide chemotaxis for macrophages, fibroblasts, and keratinocytes, respectively. The arrival of these cells marks the transition from a process of clearing and stabilizing the wound to one of proliferation and repair. This complex sequence of events leads to wound repair either in a direction of tissue necrosis with scar formation or reconstruction with return of function. The inflammatory process may take up to 14 days in a healthy wound bed and is thought to control both the quality of tissue repair and the amount of scar formation [4]. In flap failures there is a prolonged inflammatory phase with an increase of free radicals in response to ischemia that further prolongs tissue repair.

Traditional teaching in the care of ischemic wounds has revolved around creating an environment conducive to wound healing. This is done by protecting the healing wound from trauma and bacterial invasion, maintaining a moist wound surface, and removing necrotic tissue and foreign bodies which prolong the inflammatory process and act as media for bacterial growth. This has typically been done with wet to dry dressings and periodic gentle or surgical debridement of necrotic tissue.

 The extracellular matrix (ECM) contains a number of different growth factors, thought to speed the repair of difficult wounds. Additionally organized ECM scaffolding may be able to modulate the wound healing response toward native tissue orientation rather than scar tissue formation [5]. An extracellular matrix is a secreted product of cells that populate a given tissue. The composition and ultrastructure of the ECM are multifactorial and determined by the phenotype of the underlying cells, the pH of the environment, biochemical milieu, mechanical forces, and inherent gene expression patterns. Conversely, the ECM may influence the behavior and phenotype of the resident cells. The ECM mitigates cell attachment, migration, and 3D spatial arrangement [6].

Three main tissues have been investigated for ECM scaffolds including urinary bladder matrix (UBM), small intestine submucosa (SIS), and liver (LECM) [6]. Extracellular matrix contains the secreted products of the native cells to the respective tissue including collagen I, collagen IV, laminin, and fibronectin [7]. The basement membrane plays an important role in epithelial surfaces and directionality of tissue regeneration [7]. In particular the integrity of the basement membrane of UBM has been shown to be unaffected by commercial preparations used to delaminate, decellularize, disinfect, and sterilize. The presence of a basement membrane has been shown to prevent fibroblasts from invading the luminal side of the UBM scaffold [7]. A preserved basement membrane has been shown to induce cultured cells toward differentiation, migration, and organization of epithelium [8].

 This paper serves as an initial report of four patients on whom the MatriStem urinary bladder ECM (ACell, Columbia, MD, USA) was used as a salvage option of failed local and regional flaps. MatriStem regenerative medicine technology is a naturally occurring bioscaffold derived from porcine tissue. When MatriStem is placed onto a wound, it is resorbed and replaced with new native tissue where scar tissue would normally be expected. This technology gained FDA approval for use in human subjects in 2009 for wound care, soft tissue repair, and general surgery. To the best of our knowledge, this is the largest series of patients treated for flap failures of the head and neck using this technology. 

## 2. Methods

 The senior author (G. F. Zuliani) evaluated all patients after the diagnosis of flap failure was established, and reconstructive options were evaluated. Of the four patients, two were failed flaps from the senior author, and two were referrals from colleagues. Patients were evaluated and counseled on reconstructive options, and the role of extracellular matrix for tissue regeneration was discussed. Appropriate informed consent was obtained on all patients. Standard preoperative and postoperative pictures were taken, and the patients were followed on a weekly to biweekly basis until resolution of their soft tissue defect. The study was IRB approved by Wayne State University, Detroit, MI, USA.

 Patients were either taken to the operating room or more commonly underwent debridement and application in the clinic. Wound beds were debrided down to healthy bleeding tissue. On the first application, 60 mg of ground micronized porcine urinary bladder ECM was applied on the wound bed. Following this, an appropriately sized extracellular matrix plastic surgery sheet was applied to the wound with basement membrane down to ensure that epithelialization occurs on the external surface. Prior to placing the sheet on the wound bed, the sheet was tailored to the appropriate size and reconstituted in 20 cc of normal saline for fifteen minutes to ensure maximum pliability. The bioscaffold was then sutured into place with multiple interrupted 5-0 polypropylene sutures. Petroleum jelly impregnated gauze (Adaptic, Johnson and Johnson, New Brunswick, NJ, USA) and water based lubricating jelly were then applied to moisturize and protect the extracellular matrix. This was then covered with a sterile gauze dressing, Mastisol (Ferndale Labs, Ferndale, MI, USA), and a Tegaderm (3M, St. Paul, MN) dressing to ensure maximum conformity to the topography of the facial aesthetic unit being covered.

During their weekly office visits, patients' dressings were removed, and their wounds were evaluated. During office visits, photographs were taken, and patients underwent gentile debridement of the wound if grossly necrotic tissue remained. During the initial four to five weeks, a thick fibrinous and sometimes malodorant discharge could be seen overlying the wound. This layer often confused with purulence was not removed as it contains growth factors and antibacterial properties [7]. In no cases were antibiotics used on our patients during this process. The actual scaffold starts to resorb in 48 hours and continues for approximately a month to let the natural healing process proceed with minimal risk of inflammation and thus scarring [5]. If this layer was sparse and additional granulation tissue was needed, additional micromatrix and another plastic surgery sheet were applied. This was followed by the same formal dressing which would not be removed again until the next office visit. Once acceptable granulation tissue and wound contraction were complete, a thinner bioscaffold wound sheet was applied to start the epithelialization process. 

## 3. Results

### 3.1. Case  1

This is a 56-year-old male who initially presented with a large 15 × 15 cm cutaneous squamous cell malignancy on the left side of his face. He had no known significant past medical history other than a recent history of pulmonary embolism. He was taken to the OR by a head and neck oncologist for wide local resection of his tumor, left superficial parotidectomy, left lateral temporal bone resection, and left modified radical neck dissection. Reconstruction consisted of a left trapezius myocutaneous flap and left cervicofacial flap.

His postoperative course was complicated by extensive wound breakdown and abscess formation under his trapezius muscle flap, and he returned to the OR 2 weeks later for debridement, and was treated with twice daily dressing changes. On examination there was a 6 × 7 cm area of exposed cranium devoid of periosteum at the superficial aspect of his dehisced trapezius flap. After his infection cleared, he returned to the OR by the senior author, now 6 weeks status after his first surgery, for placement of scalp expanders, a pectoralis major myocutaneous flap, and placement of MatriStem over the cranium. He was discharged from the hospital two days later. His postoperative course was complicated by recurrence in the right periorbita for which he underwent adjuvant chemotherapy and external beam radiation. He is now disease free and has started to undergo additional treatments with the MatriStem bioscaffolds. At this time only ten percent of the wound necessitates granulation tissue coverage. 

### 3.2. Case  2

 This is a 5-year-old female who initially presented with a right-sided microtia and external auditory canal atresia with no significant other past medical history. She underwent a stage 1 microtia repair with left-sided synchondrosis rib grafting. Her postoperative period was complicated by pneumonia and flap congestion, which over the next week developed necrosis. This was most likely secondary to a very thin flap covering the large cartilaginous framework. Traditionally necrosis with cartilage exposure necessitates removal of the entire complex. On postoperative day 10 she returned to the OR after the senior author was consulted for debridement and possible salvage with the MatriStem bioscaffold.

 Dressings were changed on a weekly basis for the next 3 weeks. Five weeks status after the initial surgery, she returned to the operating room for a definitive advancement flap and closure and suffered no other operative complications, costal cartilage remained viable throughout her recovery. She has since undergone second stage with release of the neoauricle, and lobule transposition. 

### 3.3. Case 3

**Figure 1:**
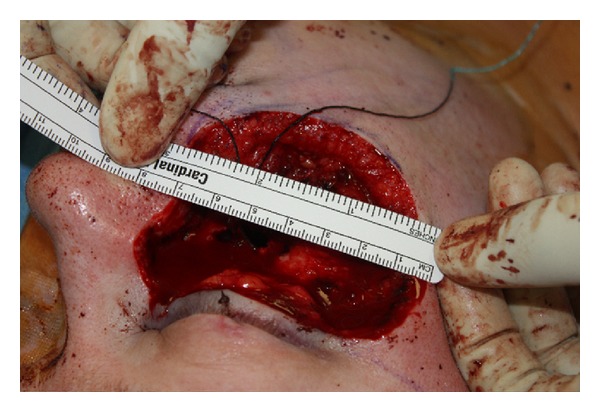
Case 3, 7 × 17 cm right-sided facial skin and maxillectomy defect after resection of right-sided squamous cell carcinoma.

 This is a 57-year-old woman with recurrent squamous cell carcinoma of the right cheek with maxillary sinus involvement. Her past medical history was significant for diabetes, hypothyroidism, asthma, and morbid obesity. She had previously had two unsuccessful Mohs surgeries and presented with recurrence on CT with a deep lesion in the cheek musculature involving the infraorbital nerve abutting the maxillary sinus. She was then taken to the OR by a head and neck oncologist for a right maxillectomy, dermal fat graft, and a large 7 × 17 cm right cervicofacial flap (Figure 1). 

**Figure 2:**
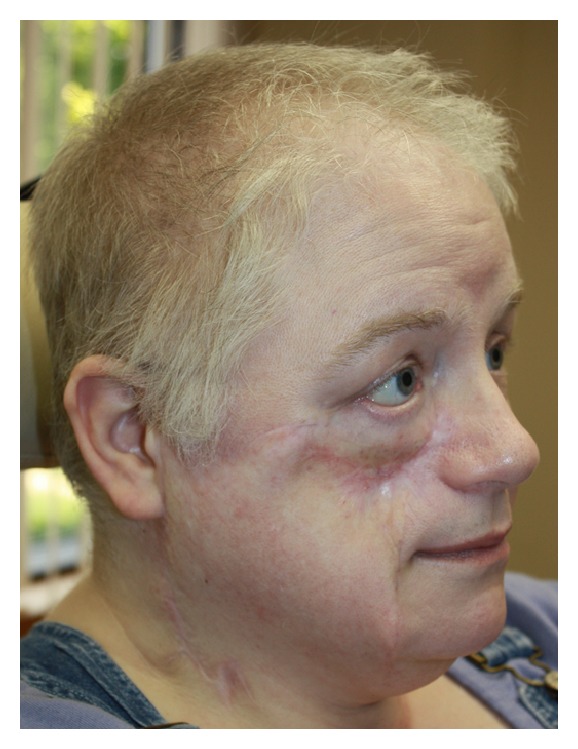
Case 3, one year after completion of external beam radiation therapy. One can see the smooth skin texture. Cheek deformity and combination of paralytic/cicatricial ectropion right lower lid are evident. Reconstruction of these deformities planned in the near future.

Within the first 24 hours of surgery, her cervicofacial flap showed a significant amount of congestion, and leech therapy was begun three times a day. This was unsuccessful, and she suffered from significant breakdown. She was discharged home, and outpatient gentle debridement with placement of MatriStem dressing was initiated on a weekly basis in the office. After 12 weeks of weekly dressing changes the wound was fully reepithelialized without complication. Her initial resection had positive margins, and she subsequently started external beam radiation therapy. Receiving a total of 60 Gy over 30 radiation treatments. She suffered no further wound breakdown, is now over one year out from her original surgery, and is free of disease (Figure 2). 

### 3.4. Case 4

**Figure 3:**
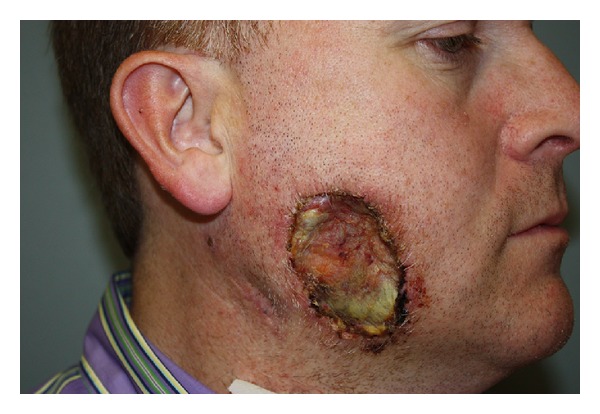
Case  4 shows 6 cm diameter right cheek defect after melanoma resection with split thickness skin graft closure.

 This is a 48-year-old male who presented with a primary dermal melanoma, (Breslow level of 14 mm, Clark level IV, no ulceration) to his right jawline. He underwent wide local excision and sentinel node biopsy for this lesion. At the time of surgery he had a 6 cm defect, which was closed with a split thickness skin graft (Figure 3). He was scheduled to receive interferon therapy but wanted to delay because of cosmetic concerns regarding the cheek and the limitation on surgical treatments while receiving this adjuvant therapy. One month after operation, he underwent a cervicofacial advancement flap for definitive closure of his facial wound. 

**Figure 4:**
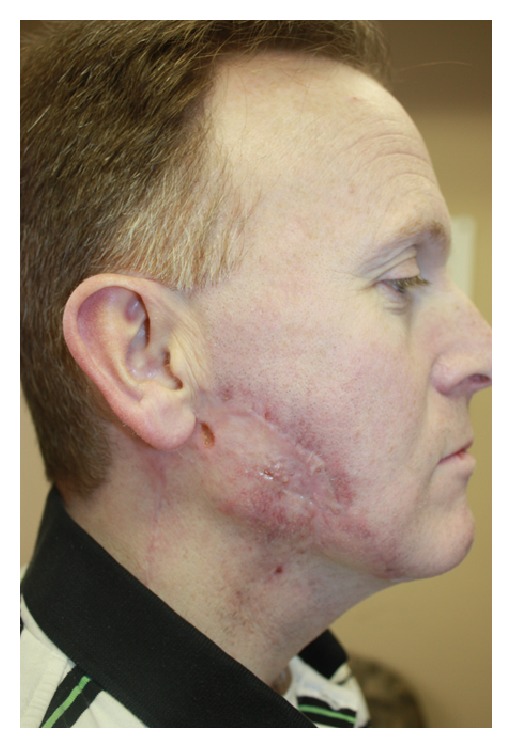
Case  4, eight weeks after initial flap reconstruction and now receiving interferon alpha-IIB therapy. Despite severe neutropenia, full epithelialization occurred within 6 weeks of MatriStem placement.

Within 2 weeks of the advancement flap he began to have breakdown at the anterior and posterior boarders of this flap and was taken for debridement with MatriStem placement in the office. MatriStem was changed based on the regimen as previously described, and his wound rapidly contracted in the first 4 weeks. Interferon treatment was delayed one week. He then began interferon alpha-IIB treatments three times a week. During this time the patient became extremely neutropenic, and during the next four weeks he had improvement to complete resolution of his wound, despite white blood cell counts being as low as 2000 cells (Figure 4). He is now 8 months out from his initial reconstructive surgery and is still receiving interferon therapy without wound breakdown. 

## 4. Discussion

 Flap failure occurs in approximately 5 percent of flaps with higher likelihood of failure in patients with a smoking history or vascular disease. Traditional methods to treat flap failures such as wet to dry dressings provide obliteration of dead space, gentle debridement with removal of necrotic and likely infected tissue, and moistening of tissue beds. However this method is slow and labor intensive as dressings likely need to be replaced twice or three times a day, and in many instances causes significant discomfort to the patient.

Application of an extracellular matrix for tissue regeneration is thought to serve to promote wound coverage by both providing bulk to the wound as well as serving as an excellent substrate for growth of autologous keratinocytes. Typically there is angiogenesis from the wound bed along with dense cellular infiltrate from the surrounding dermis and subcutaneous tissue as well as rapid keratinocyte infiltration from the wound edges [9]. This may provide an increased speed in time to wound closure, while significantly reducing hospital stay and home nursing costs as treatments can be performed weekly on an outpatient basis. Overall this can lead to a decrease in the delay to adjuvant therapy after flap failure.

 MatriStem, which is a porcine derived extracellular matrix, provides bulk to the ischemic wound as well as a substrate and growth factors to promote tissue regeneration. It is this site that directed regeneration property of MatriStem that is the main theoretical advantage over traditional wet to dry dressings. MatriStem Wound Care Matrix facilitates the healing process and is used primarily on partial and full-thickness wounds such as burns and diabetic venous and arterial and pressure ulcers. The cost of wound care for flap failures has not been specifically examined in the head and neck, but one may extrapolate the direct and indirect costs from wound care in other parts of the body. For instance, the infected foot ulcer is the most common reason for hospitalization among patients with diabetes, resulting in over 200,000 days of inpatient care. Wound care devices, home health care and hospital costs associated with wound care cost the U.S. healthcare system over $7 billion in 2007 [10]. A 1999 study from the Cleveland Clinic actually examined the direct medical costs associated with treating venous stasis ulcers in a clinical setting. The average total medical cost per patient was near $10,000, with home health care, hospitalizations, and home dressing changes accounting for 48%, 25%, and 21% of total costs, respectively [11]. These authors believe the burden to actually be much greater since indirect costs such as time away from work were not accounted for in their study.

 Funk et al. estimated that the total cost of free tissue transfer in the head and neck after 1 year of surgery and postoperative therapy was approximately $150,202 when looking at a case series of 21 patients [12]. Kroll et al. estimated the cost of surgery alone for both myocutaneous and free tissue transfer to be between $28,000 and $41,000 [13]. This is compared to approximately $8,500 for 15 weeks of weekly treatment using the largest available (10 cm × 15 cm) wound sheet and 1000 mg of matrix powder which would be a typical treatment for a very large wound. It should be noted that these costs are for primary treatment of defects as well. The decision for how to treat flap failure, either with conservative management or with additional flaps and grafts must be carefully weighed. Flap failure in a salvage setting is 4.6 times more likely with a success rate of 53.3% [14].

 MatriStem's porcine derived bladder ECM has other advantages over traditional wound dressings. Patients had to endure dressing changes on a weekly basis instead of traditional daily or twice daily dressing changes; dressing changes were less painful for the patient, as well as being less labor intensive for the care provider. Debridement at the time of dressing change was gentle and well tolerated. Because of its antibacterial properties, no oral or IV antibiotics needed to be administered. In our study, tissue regeneration occurred significantly faster than expected in our patients. Presumably this allowed those patients with a cancer diagnosis to start adjuvant therapy earlier then would have been possible with traditional methods. There are, however, no studies to date to demonstrate whether traditional dressing changes are indeed slower than using this xenograft. A blinded study examining time to full repair as well as clinical perception of scarring would be ideal, yet difficult to perform. Finding individuals to serve as their own controls with part of their wound being treated with dressing changes and the other part with the xenograft would be challenging. Comparing the two treatment modalities between patients would be full of confounding factors such as the subjectivity of analysis, medical comorbidities, and skin types which have a propensity toward hypertrophic scarring. 

 In the future, porcine derived ECM may be used intraoperatively in high-risk patients, such as those that have had previous radiation, diabetes, or other vascular disease to promote wound healing and prevent flap breakdown. Perhaps this material will allow appropriate tissue coverage in those patients not suitable for anesthesia, avoiding extensive flap reconstruction in the head and neck. In fact, this is actually being done with selected patients in our clinic right now. Additionally the ECM is now available in a gel form, which may have additional reconstructive and cosmetic uses similar to those used by a collagen filler. Extracellular matrix bioscaffolds are an emerging field in medicine and show significant promise in the treatment of chronic or ischemic wounds.
